# Supplementary material for: Prevalence of loneliness amongst older people in high-income countries: A systematic review and meta-analysis
Source: PLoS One. 2021 Jul 26;16(7):e0255088. doi: 10.1371/journal.pone.0255088 (PMC8312979; doi:10.1371/journal.pone.0255088)
Supplement: S1 File — (DOCX) [file pone.0255088.s001.docx]

## **S1 File. Medline search strategy**

| 27 | limit 26 to (english language and yr="2000 -Current") |
| --- | --- |
| 26 | 21 and 25 |
| 25 | 22 or 23 or 24 |
| 24 | ((senior* or old*) adj1 (person or people or community or population or adult*)).mp. [mp=title, abstract, original title, name of substance word, subject heading word, floating sub-heading word, keyword heading word, protocol supplementary concept word, rare disease supplementary concept word, unique identifier, synonyms] |
| 23 | (elder* or geriatric*).mp. [mp=title, abstract, original title, name of substance word, subject heading word, floating sub-heading word, keyword heading word, protocol supplementary concept word, rare disease supplementary concept word, unique identifier, synonyms] |
| 22 | aged/ or "aged, 80 and over"/ or frail elderly/ |
| 21 | 6 and 20 |
| 20 | 7 or 8 or 9 or 10 or 11 or 12 or 13 or 14 or 15 or 16 or 17 or 18 or 19 |
| 19 | ((social* or societ* or perce or person*) adj3 (isolation or isolated or alienation or alienated or relation* or detachment or detached or contact or link or tie or ties or support* or participation or activ* or engage* or connect* or disconnect* or cohesion or cohesive or embedded or vulnerab* or interect*)).ti. |
| 18 | (social wellbeing or social health or social capital).ti. |
| 17 | (lonely or loneliness or solitude).ti,ab. |
| 16 | social participation/ |
| 15 | psychosocial deprivation/ |
| 14 | friends/ |
| 13 | interpersonal relations/ |
| 12 | social distance/ |
| 11 | community networks/ |
| 10 | social support/ |
| 9 | social alienation/ |
| 8 | social isolation/ |
| 7 | loneliness/ |
| 6 | or/1-5 |
| 5 | ep.fs. |
| 4 | incidence.ti. |
| 3 | exp incidence/ |
| 2 | prevalence.ti. |
| 1 | exp prevalence/ |
